# Corrosion Resistance of a Sand Particle-Modified Enamel Coating Applied to Smooth Steel Bars

**DOI:** 10.3390/ma7096632

**Published:** 2014-09-15

**Authors:** Fujian Tang, Genda Chen, Richard K. Brow, Michael L. Koenigstein

**Affiliations:** 1 Department of Civil, Architectural and Environmental Engineering, Missouri University of Science and Technology, Rolla, MO 65401, USA; E-Mail: ftkr7@mst.edu; 2 Department of Materials Science and Engineering, Missouri University of Science and Technology, Rolla, MO 65401, USA; E-Mail: brow@mst.edu; 3 Managing Partner, Pro-Perma Engineered Coatings, Rolla, MO 65401, USA; E-Mail: mkoenigstein@pro-perma.com

**Keywords:** corrosion resistance, enamel coating, sand particles, EIS, SEM, XRD

## Abstract

The protective performance of a sand particle-modified enamel coating on reinforcing steel bars was evaluated in 3.5 wt% NaCl solution by electrochemical impedance spectroscopy (EIS). Seven percentages of sand particles by weight were investigated: 0%, 5%, 10%, 20%, 30%, 50% and 70%. The phase composition of the enamel coating and sand particles were determined with the X-ray diffraction (XRD) technique. The surface and cross-sectional morphologies of the sand particle-modified enamel coating were characterized using scanning electron microscopy (SEM). XRD tests revealed three phases of sand particles: SiO_2_, CaCO_3_ and MgCO_3_. SEM images demonstrated that the enamel coating wetted well with the sand particles. However, a weak enamel coating zone was formed around the sand particles due to concentrated air bubbles, leading to micro-cracks as hydrogen gas pressure builds up and exceeds the tensile strength of the weak zone. As a result, the addition of sand particles into the enamel coating reduced both the coating and corrosion resistances.

## 1. Introduction

The most commonly used coatings to protect steel bars from corrosion in reinforced concrete (RC) structures are fusion-bonded epoxy (FBE) and hot-dipped galvanized zinc (HDG) [1,2,3]. The main issue with epoxy-coated bars is the reduction in bond strength or force transmissibility between steel bars and their surrounding concrete [4,5]. As a result, coating imperfection and external damage would cause disbondment of the FBE coating and under-film corrosion [6]. Hot-dip galvanized zinc coatings also have two main concerns in engineering applications. First, the zinc coating corrodes vigorously due to the high alkaline environment in fresh concrete if no passive film is formed. Second, the hydrogen produced in the cathodic reaction increases the porosity of adjacent cement pastes and thus reduces the bond strength between the bars and the concrete [7].

Alternatively, porcelain enamel coatings for reinforcement steel bars have been systematically studied for both enhanced corrosion resistance and increased bond strength in concrete [8,9,10,11,12]. Specifically, three types of enamel coatings (pure, mixed and double enamel) applied on reinforcement steel bars were investigated. The pure enamel is commercially available. The mixed enamel is prepared by mixing 50% of the pure enamel by weight with 50% calcium silicate from Portland cement, which was designed to increase the bond strength between steel bars and concrete by both mechanical (roughness) and chemical (reaction) effects with the surrounding concrete. Results showed that the bond strength was increased up to 15% for coated deformed bars, which was accompanied by the decrease of corrosion resistance due to the change in coating microstructure. These results were confirmed by the observed improvement of a weak layer of transition zone near the steel bars [13]. To improve both corrosion resistance and bond strength, the double enamel was introduced with an inner layer of pure enamel and an outer layer of mixed enamel. Both the pure and double enamels had isolated air bubbles and can prevent the penetration of aggressive chemicals to the surface of steel. The addition of calcium silicate to the enamel, however, produced connected channels in the enamel coating, which provides pathways for aggressive chemicals to reach the steel.

Past studies have shown that adding sands into an epoxy coating can increase the bond strength of an epoxy-coated bar in concrete without affecting its corrosion resistance [14,15]. Therefore, an attempt was made to add sand particles into the enamel coating for simultaneous enhancement of both corrosion resistance and bond strength, since sand particles can increase the enamel coating surface roughness, which would increase the steel bar interlocking with the concrete.

This study aims to investigate the corrosion performance of sand particle-modified enamel coating in 3.5 wt% NaCl solution. Smooth steel bars were coated with enamel that is modified with sand particles of various percentages between 0% and 70% by weight. The surface and cross-sectional morphologies of these coatings were characterized with scanning electron microscopy. The corrosion performance of the coating was evaluated by electrochemical impedance spectroscopy.

## 2. Results and Discussion

### 2.1. Characterization of Enamel Coating and Sand Particles

**Figure 1:**
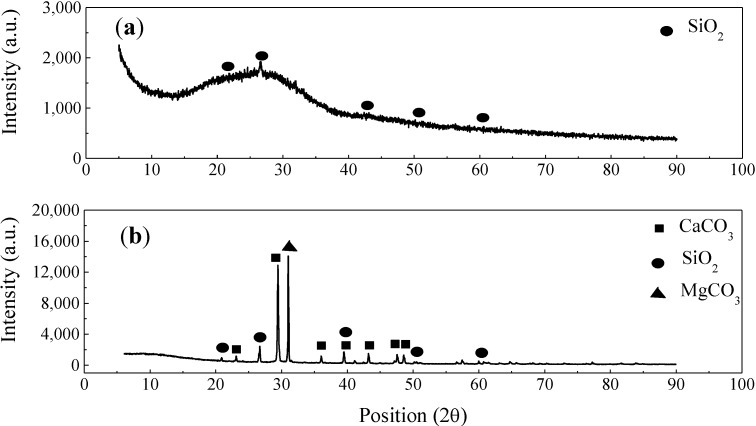
Phase compositions of (**a**) enamel coating, and (**b**) sand particles.

The phase compositions of the enamel coating and sand particles were analyzed by X-ray diffraction, as presented in Figure 1. An amorphous hump, centered near 2θ = 27°, dominates the pattern from the enamel coating, as indicated in Figure 1a. This observation is consistent with the glassy nature of borosilicate materials. Small peaks correspond to the presence of a small amount of crystalline quartz in this sample. As indicated in Figure 1b, three phases of sand particles (SiO_2_, CaCO_3_ and MgCO_3_) were observed.

**Figure 2:**
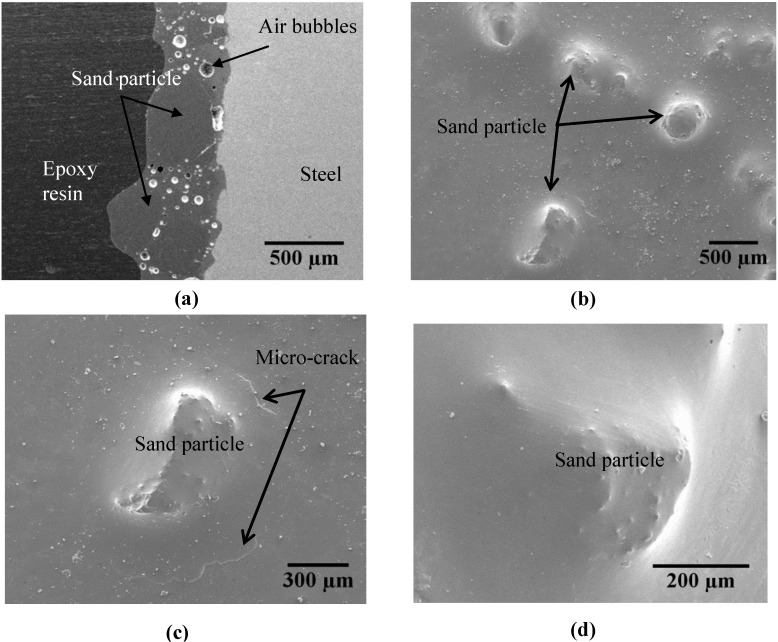
SEM images of enamel coating with 70 wt% sand particles: (**a**) steel-enamel cross-section; (**b**) enamel coating surface; (**c**) sand particle with micro-crack; and (**d**) sand particle without micro-crack.

The cross-sectional and surface morphologies of enamel coatings with different percentages of sand particles are similar. Therefore, only the enamel coating with 70 wt% sand particles was characterized with SEM and presented in Figure 2 for the cross-section of an enamel-coated smooth steel bar. As shown in Figure 2a, the coating thickness ranged from 400 to 700 μm due to the addition of the sand particles. Several small air bubbles (approximately 40 μm in diameter) are observed in the enamel coating and concentrated around the sand particles. These bubbles resulted from the chemical reaction during high temperature enameling [16,17]. They formed a weak zone around the sand particles and were locations for potential micro-cracks. As shown in Figure 2b, all of the sand particles were embedded completely underneath the enamel coating. However, a few micro-cracks were sometimes present around part of the sand particles.

Figure 2c,d demonstrates the magnified SEM images of sand particles with and without surrounding micro-cracks, respectively. The presence of micro-cracks around some sand particles is attributed to the increased porosity in the weak zone and the internal hydrogen pressure.

As observed from the XRD analysis, silicate particles (SiO_2_) are the primary constituent of sand particles, although a few carbonate particles (CaCO_3_ and MgCO_3_) are present, as well. Carbonate sand particles are decomposed chemically at high temperature [18,19]. At temperatures between 660 and 979 °C, calcium carbonate breaks down into calcium oxide, with the release of carbon dioxide, as shown in Reaction (1). Similarly, magnesium carbonate is also decomposed between 741 and 838 °C, as shown in Reaction (2). The release of carbon dioxide due to the thermal decomposition of carbonate particles, together with the air bubbles generated in the enameling process, increased the porosity in the weak zone.

CaCO_3_ → CaO + CO_2_ ↑ (1)

MgCO_3_ → MgO + CO_2_ ↑ (2)

At the enameling temperature, the water in the enamel slurry breaks down into oxygen and hydrogen. Due to its increased solubility in steel, the hydrogen diffuses into the substrate steel at high temperature. However, in the cooling stage, it diffuses out and concentrates at the enamel-steel interface, thus generating internal pressure. Depending upon the amount of hydrogen, the internal pressure at the enamel-steel interface sometimes exceeds the tensile strength of the enamel coating locally, as shown in Figure 2c. As a result, a small piece of enamel coating would flake off the steel substrate. This is the commonly observed fish scale phenomena in enamel coating. In other cases, as shown in Figure 2d, no micro-cracks were observed, likely due to insufficient hydrogen-induced pressure. However, no fish scale was ever observed in the enamel coating without sand particles, since the enamel coating itself is strong enough to resist the internal hydrogen pressure.

### 2.2. Electrochemical Impedance Spectroscopy

**Figure 3:**
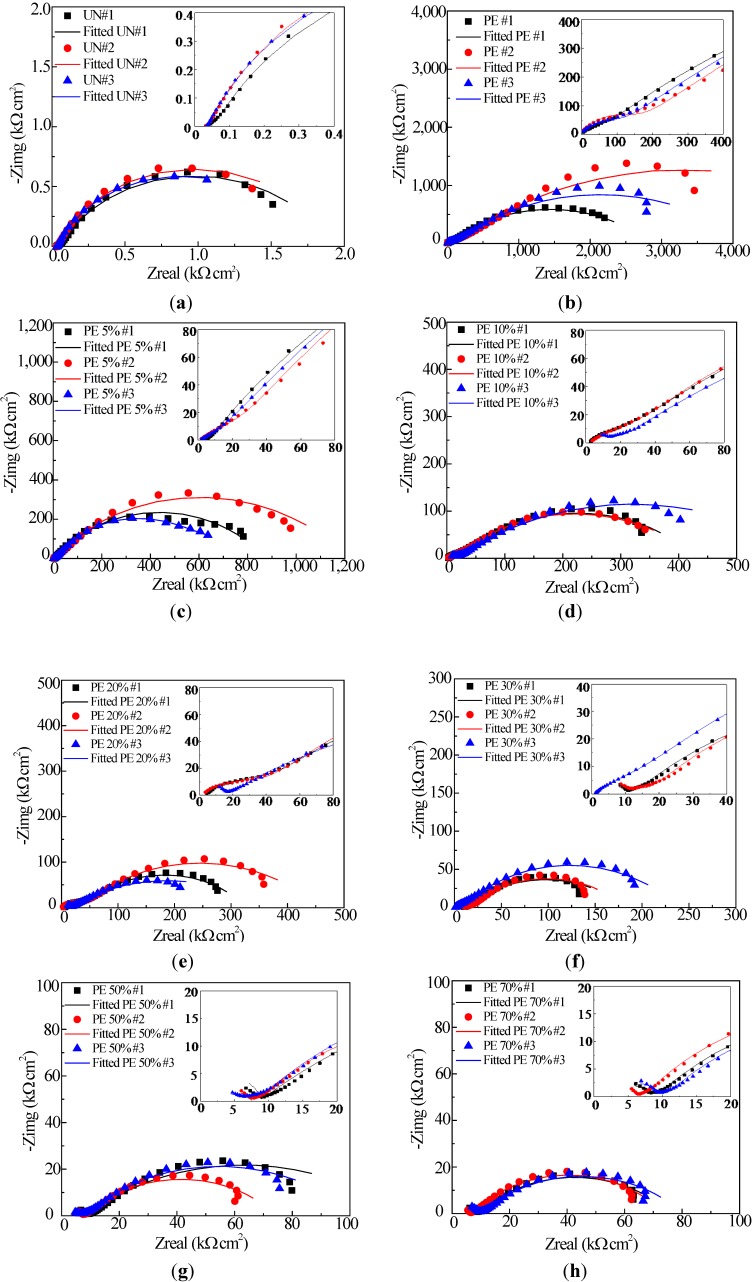
Typical EIS diagrams of smooth steel bars: (**a**) uncoated (UN); and (**b**–**h**) coated with pure enamel (PE) modified with 0%, 5%, 10%, 20%, 30%, 50% and 70% sand particles by weight.

Figure 3 shows the EIS diagrams of uncoated and coated smooth steel bars with sand particle-modified coating in the format of a Nyquist plot. For each percentage of sand particles, three samples were tested with consistent behavior. In these diagrams, the scattered symbols represent the experimental data, and the continuous lines represent the fitted results with equivalent electrical circuit (EEC) models, as will be discussed later. It can be observed from Figure 3 that one large depressed semi-circle appeared in the low frequency range for all samples, including the uncoated steel bars (Figure 3a). This semi-circle is related to the properties of steel-electrolyte interface (double-layer capacitance and charge transfer resistance). With an increase of sand particle percentage, the radius of the large semi-circles decreased significantly. For enamel-coated steel bars with different percentages of sand particles, a small depressed semi-circle appeared in the high frequency range, which is associated with the enamel coating properties (coating capacitance and resistance).

**Figure 4:**
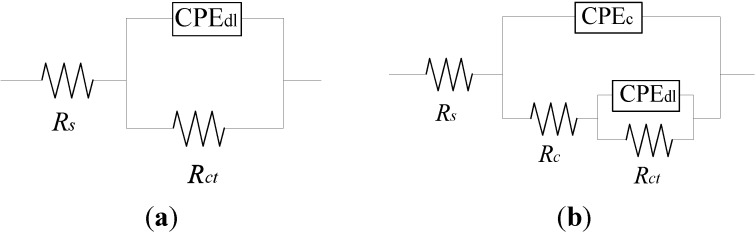
Equivalent electrical circuit model for the (**a**) uncoated and (**b**) enamel-coated steel bar. CPE, constant phase element.

As shown in Figure 4, two EEC models were used to fit the EIS test results for uncoated and enamel-coated steel bars, respectively. These two models were commonly used by other researchers to evaluate the corrosion resistance of steel samples with and without coatings [20,21,22,23,24,25]. Specifically, *R*_s_ represents the solution resistance, *R*_c_ and CPE_c_ denote the coating resistance and capacitance and *R*_ct_ and CPE_dl_ represent the charge transfer resistance and double-layer capacitance at the steel-electrolyte interface.

Replacement of capacitance with a constant phase element (CPE) in the EEC models is attributed to the non-homogeneity in the corrosion system, which is generally attributed to the distributed surface reactivity, roughness, electrode porosity and current and potential distribution associated with the electrode geometry [26]. The impedance of CPE can be represented by the following equation:

*Z_CPE_* = 1/[*Y* (*j*ω)*^n^*] (3)

where *Y* and *n* are two parameters related to the CPE. When *n* = 1, CPE resembles a capacitor with capacitance *Y*. When *n* = 0, CPE represents a resistor with resistance *Y*^−1^. The effective capacitance can be calculated from the CPE parameters in a normal distribution non-homogeneity condition [27]:

*C* = *Y*^1/*n*^*R*^(1−*n*)/*n*^ (4)

where parameters *R*_c_, *Y*_c_ and *n*_c_ were used to calculate the effective capacitance of the coatings *C*_c_; *R*_ct_, *Y*_dl_ and *n*_dl_ were used to calculate the effective capacitance of double-layer *C*_dl_, respectively.

ZSimpWin was used to fit the EIS data with the two EEC models. The chi-squared values in the fitting are in the range from 10^−4^ to 10^−3^, indicating a good fitting. The fitted results were shown in Figure 3 by the continuous line.

**Figure 5:**
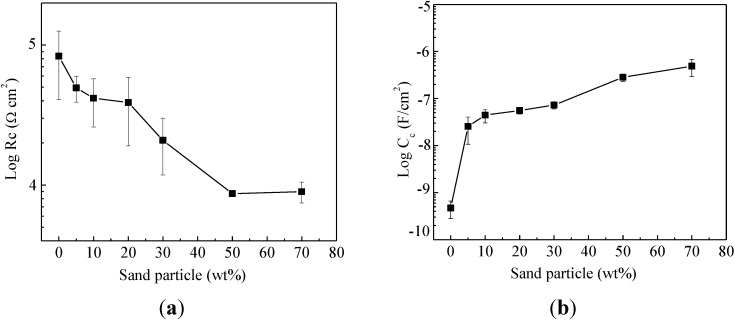
The effect of sand particle content on coating properties: (**a**) coating resistance; and (**b**) coating capacitance.

Figure 5 shows the dielectric properties of sand particle-modified enamel coatings in terms of coating resistance *R*_c_ and coating capacitance *C*_c_. Each represents the average of three samples with an error bar representing one standard deviation. In general, the coating resistance is attributed to the electrical resistance to ionic transfer through the coating pores, which reflects the anti-penetrating ability of the coating to the electrolyte [28]. It is closely related to the dielectric properties, microstructure, thickness and defects of the enamel coatings modified by sand particles. As shown in Figure 5a, the coating resistance decreased from 83.2 ± 42.5 kΩ·cm^2^ for enamel coating without sand particles to 8.98 ± 1.53 kΩ·cm^2^ for enamel coating with 70 wt% sand particles. The reduction in coating resistance is due to the presence of micro-cracks around some sand particles. The coating resistance decreased rapidly when the sand particles were lower than 50%. However, it remained stable when the sand particles were equal to or over 50%. This is because when the sand particle content reached 50%, the sand particle area over the entire surface area of enamel coating reached over 90%; the addition of more sand particles did not further change the surface morphology significantly. Therefore, the area with micro-cracks would be similar, resulting in the stabilized corrosion performance.

On the contrary, as shown in Figure 5b, the coating capacitance increased with an increase of sand particle content from 0.47 ± 0.19 nF/cm^2^ for steel bar samples without sand particles to 0.49 ± 0.19 μF/cm^2^ for steel bars with 70 wt% sand particles. The coating capacitance is directly proportional to the area of the capacitor [29]. The relatively lower capacitance for steel bars without sand particles indicates a comparatively lower exposure of steel substrate to the electrolyte at the pinholes of the enamel coating, as observed in Figure 8a. As more sand particles were added, more micro-cracks were introduced, resulting in the penetration of more NaCl solution into the coating (water uptake), and the coating capacitance increased consequently.

**Figure 6:**
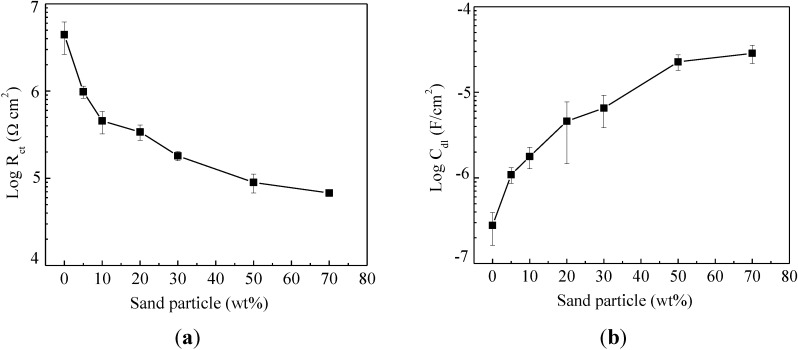
The effect of sand particle contents on: (**a**) charge transfer resistance; and (**b**) double-layer capacitance.

Figure 6 shows the evolution of charge transfer resistance *R*_ct_ and effective double-layer capacitance *C*_dl_ with an increase of sand particle content. The charge transfer resistance measures the ease of electron transfer across the metal surface, which is inversely proportional to corrosion rate [28]. As indicated in Figure 6a, the charge transfer resistance decreased from 4.42 ± 1.78 MΩ·cm^2^ for enamel-coated bars without sand particles to 67.7 ± 2.00 kΩ·cm^2^ for enamel coated bars with 70 wt% sand particles. Correspondingly, the double-layer capacitance increased from 0.28 ± 0.12 to 28.6 ± 6.8 μF/cm^2^, as indicated in Figure 6b. The decrease of charge transfer resistance and the increase of double-layer capacitance with an increase of sand particle content are attributed to the enlargement of the active corrosion area due to presence of more micro-cracks.

Compared with enamel coating modified by calcium silicate (Portland cement) in the previous study [10], the sand particle-modified enamel coating in this study has better performance in terms of both coating and corrosion properties. For the calcium silicate-modified enamel coating, the coating resistance, the coating capacitance, the charge transfer resistance and double-layer capacitance are 0.14 ± 0.05 kΩ·cm^2^, 15.2 ± 6.4 µF/cm^2^, 18.3 ± 4.6 kΩ·cm^2^ and 635 ± 184 µF/cm^2^, respectively. The addition of calcium silicate into the enamel changes its microstructure from a solid coating layer with isolated air bubbles into an amorphous and porous structure with interconnected channels [9]. Although it is 10-times higher than the corrosion resistance of uncoated bars (*R*_ct_ = 1.93 ± 0.13 kΩ·cm^2^), the corrosion resistance of steel bars with calcium silicate-modified enamel coating is 3.7-times lower than that of the steel bars coated with sand particle-modified enamel when the sand particle content reaches 70 wt%. Similarly, the coating resistance of sand particle-modified enamel with 70 wt% sand content is 64-times higher than that of the calcium silicate-modified enamel. Therefore, sand particles as an additive to enamel coating are more desirable than calcium silicate.

**Figure 7:**
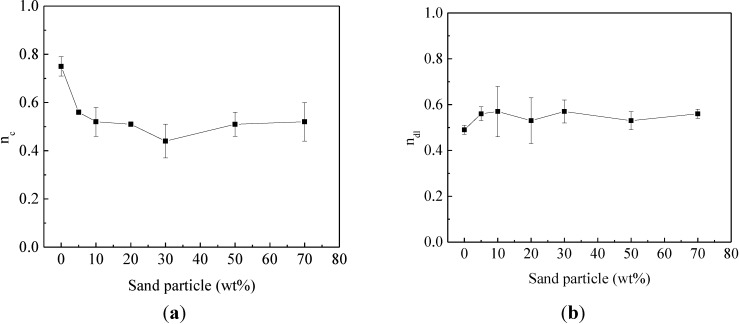
The effect of sand particle content on: (**a**) coating non-homogeneity; and (**b**) corrosion non-homogeneity.

Figure 7 shows the effect of sand particles on the non-homogeneities of both coating and a steel-electrolyte interface in terms of coating index *n*_c_ and double-layer index *n*_dl_. The coating index *n*_c_ decreased from 0.75 for steel bars without sand particles to 0.50 for steel bars with sand particles. The non-homogeneity of enamel coating without sand particles was attributed to the pinhole and coating defect generated in the enameling process. The decrease of *n*_c_ after the addition of sand particles was attributed to the presence of micro-cracks and the different types of sand particles, both of which reduced the homogeneity of the enamel coating. However, no general trend was observed for the evolution of steel-interfacial index *n*_dl_ for enamel-coated steel bars with or without sand particles. The value of *n*_dl_ fluctuated around 0.50.

### 2.3. Visual Observation and Analysis

**Figure 8:**
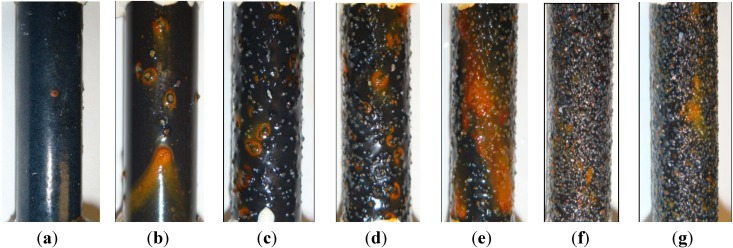
Surface conditions of enamel-coated steel bars after seven days of immersion in 3.5 wt% NaCl solution: (**a**) 0%; (**b**) 5%; (**c**) 10%; (**d**) 20%; (**e**) 30%; (**f**) 50%; and (**g**) 70% sand particles.

Figure 8 shows the surface conditions of all steel bar samples after having been immersed in 3.5 wt% NaCl solution for seven days. Most of the corrosion products accumulated around some sand particles with micro-cracks. For enamel-coated steel bars without sand particles, corrosion products could be observed at the pinholes of the coating, as shown in Figure 8a. For other enamel-coated steel bars with sand particles from Figure 8b–g, the active corrosion sites increased with an increase of sand particle content. The visually observed conditions of the enamel-coated steel bars with 50% and 70% sand particles were similar. This is because the sand particles covered most of the enamel coating area with similar active corrosion sites. This observation is consistent with the EIS results.

## 3. Experimental Section

### 3.1. Preparation of Enamel Coating and Samples

Enamel is a glass obtained by fusion at high temperature, and it may be applied to steel using either a wet or dry process. In this study, enamel coating was applied to the surface of steel bars with the wet process. Commercially-available alkali borosilicate glass frits from PEMCO (Product No. PO2025, PEMCO International, Leesburg, AL, USA) were directly used as pure enamels, and their chemical composition is 44.0 SiO_2_, 19.3 B_2_O_3_, 15.8 Na_2_O, 2.8 K_2_O, 4.7 CaF_2_, 4.6 Al_2_O_3_, 5.3 ZrO_2_, 0.9 CoO, 1.5 MnO_2_, 1.0 NiO (in weight percent) [30]. Enamel slurry was prepared by first adding 454 kg of enamel frits to 189.3 liters of water and mixing them for 20 min and, then, adding clay (31.8 kg) and borax (2.3 kg) as suspension agents and mixing again for 3.5 h. To get various sand particle contents in pure enamel, seven percentages by weight were considered: 0%, 5%, 10%, 20%, 30%, 50% and 70%.

The sand particles used are manufactured sands, whose diameter ranges from 300 to 600 μm with a median particle size of 400 μm (30–50 mesh particle size). To characterize the chemical composition, a 5.0-g sand particle was grounded into powder and analyzed with X-ray diffraction analysis to determine its phase composition.

Grade 60 smooth reinforcing steel bars with a diameter of 13 mm were used, and their chemical composition is 0.43 C, 0.22 Si, 0.95 Mn, 0.15 P, 0.07 S, 0.17 Cr, 0.03 Mo, 0.10 Ni, 0.01 Co, 0.46 Cu, 0.02 V, 0.02 Sn, and Fe balance (in weight percent). Each steel bar sample was sectioned into 76.3 mm-long pieces and then cleansed with a water-based solvent. For the coating process, the steel bar was dipped into the enamel slurry with different sand particle percentages and then heated for 2 min at 150 °C to drive off moisture. The enamel-coated steel bar was then heated in a gas-fired furnace to 810 °C for 10 min and finally cooled to room temperature. The firing treatment at high temperature melts the glass frit and fuses the enamel to the steel.

**Figure 9:**
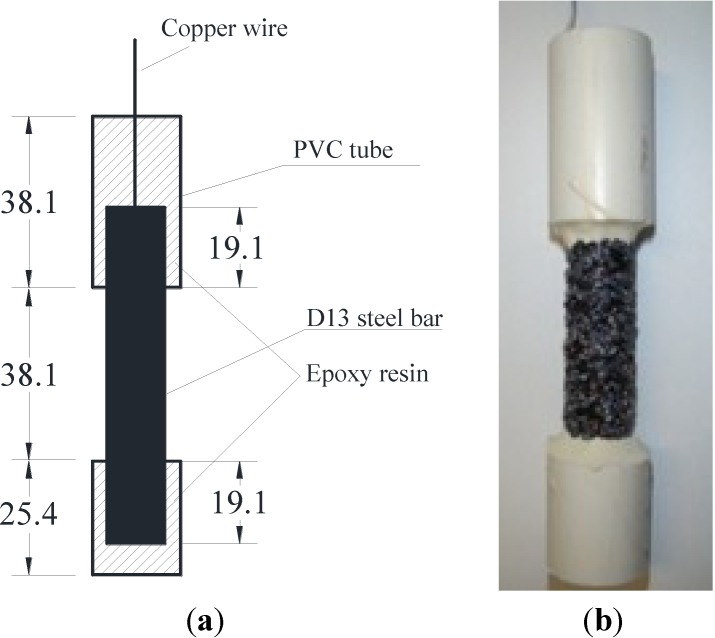
Geometry of steel bar samples: (**a**) sample dimensions; and (**b**) sample ready for test (unit: mm).

After coating, one end of the steel bar sample was first polished to expose the substrate steel on which a copper wire was welded for the corrosion test setup. PVC tubes containing epoxy resin were then used to cover the two ends in order to limit the middle portion of the steel bar being exposed to the corrosive environment, as shown in Figure 9a. Therefore, the actual length of the steel potentially exposed to the corrosive environment was approximately 38.1 mm long, and the surface area was approximately 15.2 cm^2^. A representative steel bar sample with 70 wt% sand particle-modified enamel coating is shown in Figure 9b. Steel bar samples without enamel coting were also prepared for comparison. Three identical samples were prepared for each condition to ensure the repeatability of test results.

### 3.2. Characterization of Enamel Coating with Sand Particles

The microstructures of enamel coatings were investigated with scanning electron microscopy (SEM, Hitachi S4700, Hitachi Ltd., Tokyo, Japan). Two types of enamel-coated steel bar with 70 wt% sand particles were prepared to understand both the surface and the cross-sectional morphologies with SEM images. For the observation of surface morphologies, a 3.0 cm-long steel bar was used. For the observation of the cross-section, a 1.0 cm-long steel bar was sliced from the bar with 70 wt% sand particles. These samples were cold-mounted in epoxy and ground with silicon carbide papers with grits of 80, 180, 320, 600, 800 and 1200. The samples were rinsed with deionized water, cleansed with acetone and finally dried in air at room temperature. To avoid sample charging, a carbon coating was applied prior to SEM analysis.

### 3.3. Electrochemical Tests

All samples were immersed to 3.5 wt% NaCl solution. The solution was made by mixing purified sodium chloride with deionized water. The pH of the solution was 5.72 at room temperature. A typical three-electrode set-up was used for the EIS tests. A 25.4 mm × 25.4 mm × 0.254 mm platinum sheet functioned as a counter electrode, a saturated calomel electrode (SCE) as a reference electrode and the steel bar sample as the working electrode. All three electrodes were connected to a Gamry (Gamry Instruments, Warminster, PA, USA). Reference 600 potentiostat/galvanostat with zero resistance ammeter (ZRA), for data acquisition. The tests were conducted at five points per decade around the open-circuit potential *E*_ocp_ with a sinusoidal potential wave of 10 mV in amplitude and frequency ranging from 100 kHz to 5 MHz. After 7 days of immersion tests, all samples were taken out of the solution for visual observation of the corroded surface.

## 4. Conclusions

The corrosion resistance of steel bars with enamel coatings modified by different percentages of sand particles was tested in 3.5 wt% NaCl solution by EIS. The phase composition and microstructure were characterized with X-ray diffraction and scanning electron microscopy, respectively. Based on the test results and data analysis, the following conclusions can be drawn:

(1) Enamel coating wetted well with sand particles and steel substrates. However, a weak zone was formed around the sand particles due to concentrated air bubbles.
(2) The presence of micro-cracks around some sand particles was mainly because the internal pressure of released hydrogen gas exceeded the tensile strength of the enamel coating in the weak zone.
(3) The addition of sand particles reduced both the coating resistance and the corrosion resistance of enamel-coated steel bars. The corrosion resistance of enamel-coated steel bars first decreased rapidly with the increasing percentage of sand particles and then remained stable after 50% sand particles by weight have been added.
